# Detection of *Neisseria meningitidis* in a paediatric patient with septic arthritis using multiplexed diagnostic PCR targeting meningitis/encephalitis (ME)

**DOI:** 10.1186/s12941-018-0268-7

**Published:** 2018-03-23

**Authors:** Donnchadh O’Sullivan, Barry Linnane, Amanda Mostyn, Nteimam Jonathan, Marie Lenihan, Nuala H. O’Connell, Colum P. Dunne

**Affiliations:** 1 Department of Clinical Microbiology, University Hospital Limerick, Dooradoyle, Limerick, Ireland; 2 Department of Paediatrics, University Hospital Limerick, Dooradoyle, Limerick, Ireland; 3 Centre for Interventions in Infection, Inflammation & Immunity (4i) and Graduate Entry Medical School, University of Limerick, Limerick, Ireland

**Keywords:** *Neisseria meningitidis*, Septicemia, Meningococcal, Arthritis, Multiplexed, PCR

## Abstract

**Background:**

*Neisseria meningitidis* is associated with meningitis and septicemia. Septic meningococcal arthritis is relatively uncommon and its diagnosis associated with clinical and microbiological challenges. Early recognition and treatment is required to prevent joint destruction.

**Purpose:**

We describe a case of an eleven-year-old boy with septic arthritis and the first reported use of a multiplexed diagnostic PCR test, capable of simultaneous rapid detection of 14 pathogens directly from CSF samples, to determine presence of *N. meningitides* in a synovial fluid sample.

**Results:**

In this case, blood cultures and an aspiration of the joint fluid were negative for microbial growth, but leucocytes were present. Analysis of samples using the multiplexed FilmArray^®^ meningitis/encephalitis panel (MEP) proved positive for *N. meningitidis*. In parallel, samples forwarded to an accredited reference laboratory confirmed the findings by bacterial 16S rRNA gene amplification and sequencing. Subsequent to these results, empiric treatment with intravenous flucloxacillin was discontinued and oral amoxicillin administered for 1 month. The status of the patient improved with etiology-based antimicrobial therapy.

**Conclusions:**

This case demonstrates difficulties associated with clinical and microbiological diagnosis of primary septic meningococcal arthritis. We describe the first successful use of the FilmArray^®^ MEP assay in detection of *N. meningitidis* in that context.

## Background

*Neisseria meningitidis* is associated with meningitis and septicaemia. Septic arthritis due to *N. meningitidis* is relatively uncommon. However, 11% of cases of meningococcemia have concomitant septic arthritis [1]. The most common cause of septic arthritis is *Staphylococcus aureus*, which accounts for 44% of cases [2]. Less common pathogens include *Escherichia coli* and *Pseudomonas*. *N. gonorrhoeae* is a recognised cause of septic arthritis in young adults. *N. meningitidis* has a tendency to cause oligo-articular joint infections making it difficult to differentiate from disseminated gonococcal disease [3–6].

Septic arthritis represents a medical emergency. Early recognition and treatment is required to prevent joint destruction. Typically, patients present with fever and warm, swollen, tender joints. Most commonly, the knee is involved [7]. Clinical investigation of septic arthritis includes routine analytical blood tests, arthrocentesis, and blood cultures. It is recommended that synovial fluid should be cultured, Gram-stained, and analyzed for cell count to aid initial management [8, 9].

Diagnosis is often difficult. *N. meningitidis* and *N. gonorrhoeae, which may be present in knee aspirates, appear* morphologically indistinguishable under microscopy, and cultures results may be confounded due to any prior antibiotics given [7]. In such cases, polymerase chain reaction (PCR) is used to provide a specific diagnosis.

The FilmArray^®^ meningitis/encephalitis panel (MEP) (BioFire Diagnostics LLC, Salt Lake City, UT, USA) is a in vitro diagnostic multiplexed PCR test for the simultaneous detection and identification of up to 14 bacterial, viral, and yeast pathogens directly from cerebrospinal fluid (CSF). These pathogens include; *Escherichia coli K1*, *Haemophilus influenzae*, *Listeria monocytogenes*, *Neisseria meningitidis*, *Streptococcus pneumoniae*, *Streptococcus agalactiae*, cytomegalovirus, enterovirus, herpes simplex virus 1 and 2, human herpesvirus 6, human parechovirus, varicella-zoster virus, and *Cryptococcus neoformans*/*Cryptococcus gattii.* We describe the first use of this technology, outside of its licensed use, to determine the causative agent of primary meningococcal septic arthritis in synovial fluid, in a pediatric case.

## Case

An eleven year old boy presented to the Emergency Department with a 1 day history of pain and swelling of the right knee with pyrexia on a background of Attention Deficit Hyperactivity Disorder and Autistic Spectrum Disorder. He had an antalgic gait and was unable to bear weight on it. He was fully vaccinated, had no known drug allergies and had no history of contact with other sick children. He had experienced a flu-like illness the week prior while in the United States. He had not been camping and there was no history of trauma.

On examination, the patient was stable with no evidence of systemic sepsis. Temperature was 39.2 °C. There were no skin rash or meningeal symptoms. Examination of the knee revealed a warm, swollen joint with a slight effusion and tenderness on palpation, especially on the medial aspect. Passive movements were limited secondary to pain. He was, however, able to bear weight when walking on his toes. A full examination of all systems and other joints was normal. The peripheral white cell count (WCC) was 19.3  ×  10^3^, neutrophils 16.6 ×  10^3^, erythrocyte sedimentation rate (ESR)   = 7 mm/1 h, C-reactive protein (CRP) = 78 mg/l. Blood cultures were performed and were negative following 5 days of incubation. X-rays of the right knee and both hips did not reveal any bone lesions.

Knee fluid was aspirated, whereby it appeared cloudy and yellow. Microscopic examination demonstrated absence of uric acid crystals. Leukocyte count was > 200 per high-powered field (Polymorphs 70%, mononuclears 30%). There were no organisms observed using Gram stain and culturing showed no growth. Empiric treatment for 5 days with 1 g QDS intravenous flucloxacillin resulted in eradication of pain on extension and only slight pain on maximal flexion. Limping markedly improved and the elevated temperature had resolved.

Due to clinical features of septic arthritis despite negative blood and synovial fluid cultures, a sample of the synovial fluid was forwarded to Micropathology Ltd UK for bacterial 16S rRNA sequencing. Our group has previously championed the expanded use locally of molecular microbiology techniques [10] and so, in parallel, we tested the synovial fluid using the FilmArray^®^ meningitis/encephalitis panel (MEP) despite the fact that hitherto it had been validated for testing CSF only. The test proved positive for *N. meningitidis* and was confirmed subsequently by the 16s rRNA sequencing performed by the reference laboratory, which involved both enzymatic and physical lysis of the specimen (Qiagen, Qiasymphony), PCR using broad range primers on a real-time PCR instrument (Magnetic Induction Cycler, Biomolecular Systems) and Sanger Sequencing.

As a result, the patient’s empiric treatment was revised to 1 g TDS PO amoxicillin for 1 month. The public health department was contacted and close contacts received appropriate antimicrobial prophylaxis. We evaluated the patient as an outpatient every 2 weeks for a total of 3 months. Full Blood Count, ESR, and CRP all remained within normal limits and he had made a full recovery without any joint sequelae.

## Discussion

Advances in molecular technologies for rapid species identification and susceptibility testing are mitigating protracted incubation times associated with conventional microbiology. This facilitates quicker diagnosis and a reduction in exposure of patients to empiric therapy in favor of targeted antimicrobial use [10]. This case report illustrates, for the first time, the use of the FilmArray^®^ MEP, validated for CSF samples but untried for synovial joint fluid, for the detection of *N. meningitidis*. In doing so, the difficulties associated with clinical and microbiological diagnosis of primary septic meningococcal arthritis are highlighted and the potential value of this emerging technology to aid in this challenging setting is demonstrated effectively.

Complementing conventional microbiology techniques, PCR has frequently been utilised as the primary diagnostic methodology for pathogen identification. In this case, there was a recognition that morphologically indistinguishable bacteria, i.e., causative agents of gonococcal arthritis (*N. gonorrhoeae*) or meningococcal arthritis (*N. meningitidis*), may be involved. Due to the risk of joint damage for the child, it was decided to attempt identification using the FilmArray^®^ Meningitis/Encephalitis (ME) Panel. Albeit that this application was outside of the approved license, there was a possibility of quicker, more sensitive and more specific results than Gram stain, culture, antigen detection, and 16s sequencing (at a remote laboratory).

In October 2015, the United States Food and Drug Administration approved use of the FilmArray^®^ Meningitis/Encephalitis (ME) Panel for the detection of CNS pathogens. There was acknowledgement that the technology had limitations, specifically regarding risk of false negative results when the numbers of microbial cells in the specimen are below the effective limit of detection. Indeed, due to poor accessibility to suitable clinical cases, the performance characteristics for *Escherichia coli*, *Haemophilus influenzae*, *Listeria monocytogenes*, *Streptococcus agalactiae*, and *N. meningitides* were established primarily using contrived clinical specimens. Nonetheless, in 2016, Leber et al. reported the positive results of a large-scale evaluation of 1560 prospectively collected CSF specimens, with performance compared to culture and PCR techniques [11]. However *N. meningitis* was not included in the study.

## Conclusion

Large multiplexed panels represent a paradigm shift for medical microbiology and clinical management of infectious diseases. However, more real-world evidence is needed to validate their use across a range of clinical scenarios. Therefore, this case provides useful additional new knowledge by describing, for the first time, use of this FilmArray^®^ to analyse synovial fluid rather than CSF. The accurate identification of *N. meningitis* led to enhanced care of a child at risk of joint damage, and suggests potential for the use of this technology in septic joint pathology following further validation at appropriate scale and involving multiple clinical scenarios.
